# The Theory of Planned Behaviour in Medical Tourism: International Comparison in the Young Consumer Segment

**DOI:** 10.3390/ijerph17051626

**Published:** 2020-03-03

**Authors:** Monika Boguszewicz-Kreft, Sylwia Kuczamer-Kłopotowska, Arkadiusz Kozłowski, Ali Ayci, Mohammd Abuhashesh

**Affiliations:** 1 Department of Marketing, Faculty of Finance and Management, WSB University in Gdańsk, 80-123 Gdańsk, Poland; 2 Department of Marketing, Faculty of Management, University of Gdańsk, 81-824 Sopot, Poland; sylwia.kuczamer-klopotowska@ug.edu.pl; 3 Department of Statistics, Faculty of Management, University of Gdańsk, 81-824 Sopot, Poland; arkadiusz.kozlowski@ug.edu.pl; 4 Small and Medium Enterprises Development Organization of Turkey, Ankara 06050, Turkey; aliayci@gmail.com; 5 E-Marketing and Social Media Department, Princess Sumaya University for Technology (PSUT), Amman 11941, Jordan; m.abuhashesh@psut.edu.jo

**Keywords:** medical tourism, Theory of Planned Behaviour (TPB), travel intention, Jordan, Poland, Turkey

## Abstract

The Theory of Planned Behaviour (TPB) assumes the possibility of predicting and explaining humans’ behaviour by identifying their intentions. The intentions are shaped by three groups of factors: attitudes towards, social norms and perceived behavioural control over the behaviour. The aim of the research is to examine the applicability of the TPB in medical tourism and to check whether there are differences in predicting the intentions of medical tourists from different countries. The study covered potential medical tourists—521 young consumers from three regionally important markets in medical tourism services: Jordan, Poland and Turkey. The study used a research survey to collect data, which were analysed using the multiple regression and analysis of variance methods. The research showed that the TPB model can be used in medical tourism. The results also show that the consumers’ country of origin is a significant factor when predicting their intention to use medical tourism services.

## 1. Introduction

Researchers have long struggled to explain the sources and the mechanisms of human behaviours, as well as the related decision-making processes, in order to anticipate, influence, and change the way people act. Although it has so far been impossible to come up with a universal theory explaining the mechanism, some success has been achieved in the field, a case in point being the Theory of Planned Behaviour (TPB) developed by Ajzen in 1991 [1] which is one of the most popular theories in psychology. It is an extension of the Theory of Reason Action [2]. The TPB is based on the assumption that it is possible to predict and explain human behaviour through the identification of people’s intentions. Such intentions, Ajzen claims in turn, are shaped by three factor groups, namely attitudes towards behaviours, subjective norms, and perceived behavioural control over behaviours.

Based on the aforementioned theory, numerous analyses have been conducted in various research areas including different aspects of tourism, e.g., intentions related to: travel [3,4], travel destination choice [5], type of tourism [6], choice of travel mode [7,8], environmentally responsible behaviours [9,10,11] online travel purchasing [12], and negative word-of-mouth communication in the case of restaurants [13].

The TPB has been also applied in research into health-related behaviours [14,15,16], e.g., healthy eating [17,18], pro-health behaviours [18,19,20,21], physicians’ acceptance of telemedicine technology [22], substance use [23,24], and the use of medical services [25].

The results of the research so far have shown that the TPB is an evidence-based and rigorously scrutinised theoretical framework for analysing goal-oriented human behaviour [26]. Therefore, the theory has been used as the research framework in this study of the intention to purchase medical tourism services.

The analysis of the literature available in circulation worldwide identified nine articles on applying the TPB to research on medical tourism [1]. Six of them concentrated on the results of research, conducted among foreign respondents, on the intention to purchase medical services in the reception country. In principle, these dealt with the situation on the markets of East Asia and South-East Asia, including Japanese tourists intending to use medical services in South Korea [27] and the intentions of hospitalised foreign patients (mainly from China and Russia) to visit South Korea again for medical treatment [28]; the intention of foreign tourists (mainly from Asian countries) to use medical services in Malaysia [29,30], and the intentions pertaining to the choice of destination by foreign patients using medical services in Malaysian hospitals [31]—also compliant with Sharia law in terms of health care for patients mainly from Asia and Africa [32].

Three articles presented the findings of the research conducted among respondents from sending countries in North America as to their intention to use medical services abroad. The papers concentrated on their willingness to consider medical tourism [33] and the development of a scale to predict the intention to use medical services abroad among US citizens [26], as well as on studies conducted in the USA and Canada on the extension and verification of the scale [34].

It can therefore be seen that no research exists pertaining to the use of the TPB in studies conducted in Europe and West Asia, nor do comparative analyses covering different countries. The authors of this article aim to fill the gap, presenting research conducted in Poland, Jordan, and Turkey. The selection of countries for the subject study was based on the fact that those countries are considered to be significant medical tourism markets in their respective regions [35,36,37]. Therefore, it could be assumed that respondents from these countries would be more aware of the existence of medical tourism than tourists from countries where such services are provided relatively rarely.

The aim of this article is to study the applicability of the TPB to medical tourism and to find possible differences in predicting the intentions of medical tourists from different countries. The research is concentrated on the young consumer segment, selected among students from several universities in different countries. Due to their young age, this consumer group seems potentially less interested in such services at the moment. However, with their education, fluency in foreign languages, and high economic status, they may account for a significant portion of medical tourism service users in the future. The specific research questions we would like to answer are as follows: What is the general outlook on medical tourism in the studied population? What influences the behavioural intension to participate in medical tourism (what is the direction and strength of this influence)? and finally, whether country of origin differentiates the answers to previous questions. Our research does not involve the analysis of actual behaviour or its prediction. Due to the young age of the respondents, who usually do not yet have specific diseases, the survey asked about their attitude towards foreign medical services in general, without specifying their types.

Identifying intentions related to the MT, which are influenced by attitudes and other impact factors (subjective norms and perceived behavioural control), is important for potential service providers wishing to attract clients, as well as institutions responsible for shaping health policy in the country.

The article begins with an overview of the term ‘medical tourism’ and presenting the gap in the research into the variables influencing the behavioural intentions of medical tourists. Subsequently, the essence of the TPB is discussed with its application in the area of medical tourism. Upon introducing the research model, hypothesis, and methodology, the findings of the data analysis and a discussion thereof are presented. The article concludes with some comments from the authors, as well as the identification of the limitations of the research process and the possibilities for further study in terms of applying the TPB to medical tourism.

## 2. Literature Review

### 2.1. Medical Tourism—Term and Research Gap

Medical tourism is one of the main forms of health tourism [38]. In the literature, we can find various definitions of and perspectives on medical tourism [39,40,41,42,43,44,45,46,47,48]. In this article, the following definition is accepted as the most comprehensive, as well as including the crucial reason for undertaking such travels: “Medical tourism means purposeful travelling to a foreign country to undergo intended medical treatment in order to save good health, to improve quality of life or a patient’s appearance. It is caused by financial or qualitative reasons or because of inaccessibility of particular services in patients’ countries (it may result from the lack of medical staff, lack of required knowledge, equipment, procedures or long queues to obtain the required medical treatment, and also from legal restrictions). Medical tourism frequently involves sightseeing of the visited places” [49].

The last 20 years have witnessed a considerable increase in the number of people going abroad to undergo medical procedures [35], followed by increased attention from researchers [50]. Because of its dynamics and magnitude, the phenomenon has not been thoroughly studied so far [43,45,51,52,53,54,55,56,57]. Moreover, the vast majority of the analyses have concentrated on consumers in the United States of America, without studying the situation in Europe [51]. An asymmetry can also be seen in the analyses of supply and demand in medical tourism, as less research has been dedicated to the demand aspect of the phenomenon, that is, the perspectives of actual and potential medical tourists [3,36,47,52,56,57,58,59,60,61,62,63,64,65,66]. In particular, there is little primary research into the phenomenon [34,43].

The authors emphasise the fact that very few studies have so far been dedicated to the variables influencing the intentions of medical tourists and the way they gain knowledge [30], pointing to the scarcity of empirical studies as the main problem [29]. Moreover, the geography of the research is asymmetrical – the bulk of it concentrates on Asian markets and respondents from Asia, without much analysis of other markets (e.g., the American market) [33].

### 2.2. Theory of Planned Behaviour

When introducing the TPB model, Ajzen (1991) claimed that intentions—a consequence of the interplay of specific factors—are a highly accurate predictor of various behaviours [1]. From his perspective, intentions include motivation showing to what extent people are willing to behave in a certain way and how much effort they put into behaviour. The stronger the intention to engage in a behaviour, the more probable it is the plan will be executed. Ajzen emphasises the fact that the mechanism works only when an individual is convinced that they will be free to choose their behaviours in given circumstances (i.e., they will have appropriate resources and abilities), that is, the circumstances will be under their behavioural control.

Intentions are influenced by three factors [1]. The first one is an attitude towards a behaviour, meaning its positive or negative assessment by a person. The second determinant is subjective norms, defined as the social pressure perceived by a given individual exerted by persons important to him as to whether or not to engage in a given behaviour. The third factor is perceived behavioural control, that is, an individual’s perceived ease or difficulty of performing the particular behaviour resulting from the previous experiences of the individual and the obstacles expected. Ajzen claims that the influence of each of the factors on intentions may vary depending on behaviours and situations.

According to the TPB, it is therefore assumed that people will behave in a certain way if they are convinced of a specific, and beneficial, result; if people close to them appreciate and accept their behaviour; and if they are sure they have the necessary resources, abilities, and opportunities to behave in a certain way [5].

### 2.3. Overview of Literature on TPB in Medical Tourism

Although the TPB has been applied to health-related behaviours, is it rarely used in research into medical tourism. The overview of the literature worldwide identified nine analyses of the topic, six of which concentrated on the intention of foreign respondents to use medical services in a reception country.

Lee et al. analysed the applicability of the TPB to studying Japanese tourists travelling to Korea as regards their intention to use medical tourism services [27]. The authors studied health and beautification treatments separately. The findings confirmed the fact that attitudes, subjective norms, and perceived behavioural control are predictors of the intentions of medical tourists. They noted, however, the differences in the weights of those factors in relation to particular types of services, namely health and beautification services.

Seow et al. studied the intention of foreign tourists in Malaysia to use medical services [29,30]. Their findings show significant connections between attitudes and subjective norms and intentions. However, there were no such links between behavioural control and intentions [29]. Looking for more variables to explain the medical tourists’ behaviour, the authors found out that the perception of risks and benefits (measured by the quality of the medical services in the destination country) is significantly connected with attitudes, whereas the availability of the resources (defined as easy access to technological resources and infrastructure, mostly online) relates to perceived behavioural control [30].

Park et al. studied the intentions of tourists (mostly Russian and Chinese) undergoing treatments in South Korea to use medical services abroad for the second time [28]. The authors studied the links between price awareness and health awareness, and the satisfaction with medical services and the satisfaction with travel services, followed by an analysis of the relationship between both types of satisfaction and attitude. The findings confirmed the relationship between positive attitudes towards medical tourism and the intention to use medical services abroad for the second time.

Suki et al. presented a concept of studies using the TPB for studying the intentions pertaining to the choice of destination by medical tourists visiting Malaysia [31]. Its application was presented in their subsequent publication on the factors influencing the choice of destinations where tourists can have access to health care compliant with Sharia law [32]. Their aim was to confirm the applicability of the TPB to studying the intentions of tourists looking for such services in Malaysia. The findings show that it is only attitude and perceived behavioural control that have a significant effect on the choice of the destination. Subjective norms, however, do not have such an effect. Moreover, other research showed a significant connection between piousness and attitude, and the image of a destination and subjective norms [32].

Three studies were conducted among respondents from sending countries in the context of their intention to use medical services abroad. Reddy et al. applied the TPB in an attempt to learn whether American students would be willing to consider using medical services abroad [33]. The study included additional questions regarding the nature/severity of the health condition/medical procedure, the cost of the procedure and the status of the destination (developing/third-world versus developed). The findings showed that the students had an ambivalent attitude towards medical tourism, that is, a lack of either a positive intention or strong aversion [33]. Martin et al. developed a scale based on the TPB and meant to measure the tendency of customers to participate in medical tourism, testing it on American students [26]. The scale was then replicated and verified in a subsequent study conducted among respondents from various cities in the US and Canada [34]. The findings of the study confirmed the lack of a positive attitude towards medical tourism in the US.

## 3. Research Model and Hypotheses

The studies based on the TPB have so far shown the relationship between intentions and the willingness to participate in medical tourism. However, they were sparse and focused on only a few geographical areas. This study presents intentions as regards medical tourism in subjects from Poland, Jordan, and Turkey, i.e., countries which have not been analysed in the literature, as far as the authors are aware.

**Figure 1:**
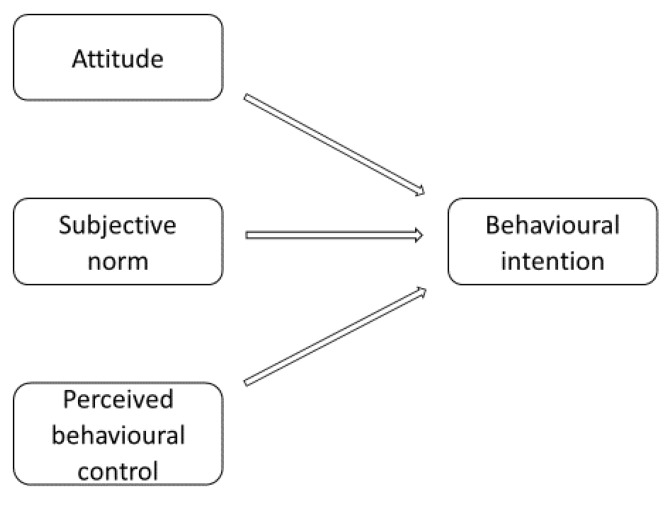
Research model. Source: Ajzen [1].

The research framework is based on the basic model proposed by Ajzen in 1991 in which intentions influence volitional behaviours, and in which the intentions are the function of the attitude towards given behaviours, subjective norms, and perceived behavioural control Figure 1 [1].

An attitude toward a behaviour is defined as a comprehensive evaluation of the behaviour. It consists of the belief regarding the consequences of the behaviour and the positive or negative evaluation of the consequences [14]. For instance, using medical services abroad will shorten the waiting time and will be beneficial to health.

**Hypothesis** **1.**  *There is a relationship between the attitude towards participating in medical tourism and the intention to participate/consider participation in medical tourism.*

The term ‘subjective norms’ refers to individuals’ perception of how their behaviour will be judged by those important to them and how important this judgment is for such an individual [1]. The norms may be of social and societal nature [31]. The first ones are opinions from family, friends, and peers; the second ones refer to a broader context of society and media. As medical services are important to our health, the opinions of both groups may be relevant for an individual. In the social context, friends and relatives may provide support for the decision to go or possibly accompany a patient; in the societal context, doctors and nurses are the authorities who may influence patients’ decisions. As far as the media goes, access to the Internet currently makes it possible for an individual to search for information on medical tourism and participate in chat groups, influencing their opinions.

**Hypothesis** **2.**  *There is a relationship between subjective norms and the intention to participate/consider participation in medical tourism.*

Perceived behavioural control refers to a sense of freedom (ease/difficulty) to behave in a certain way in a person’s own eyes [1]. It is connected with the perception of having the necessary resources and opportunities to undertake an activity and the evaluation of external conditions which one cannot fully control. The perception can result from either previous experiences of patients or their friends, or be second-hand knowledge [1]. In the case of potential medical tourists, it can also have to do with the knowledge of other languages, transport availability, or the risks connected with travel.

**Hypothesis** **3.**  *There is a relationship between perceived behavioural control and the intention to participate/consider participation in medical tourism.*

Existing research shows that attitudes towards foreign goods and services differ among consumers from different countries depending on cultural factors (ethnocentrism, aversion to a given country, stereotypes, dimensions of national cultures), demographic factors and the level of economic development of the consumer country [67,68,69,70,71,72,73,74,75,76,77]. The difference has been also shown in a study of respondents from Germany, Lithuania, and Poland as regards the dimensions of country of origin (COO) offering medical services [78].

**Hypothesis** **4.**  *The impact of individual factors in the TPB model on behavioural intentions in medical tourism differs among consumers from different countries.*

## 4. Research Method

The study of the application of the TPB model to medical tourism among young consumers was based on primary research using quantitative surveys. The tool used was a fully structured questionnaire in three languages, adjusted to the respondents’ nationality and language proficiency level.

The statistical population in the study were university-level students in Jordan, Turkey, and Poland. The sample of respondents was selected as a convenience sample of student groups. There were 521 (voluntary and anonymous) subjects in total: 183 from Jordan, 125 from Turkey, and 213 from Poland.

The survey assessed the components of the TPB. All the items in the model were measured on a 7-point Likert scale. The answers were coded as whole numbers from −3 to + 3, i.e., with a middle value of 0. The intention to consider medical tourism was measured with the item: ‘If travel abroad to obtain medical treatment were possible, I would consider it” (−3 = definitely no, +3 = definitely yes). The direct attitude score was computed as the mean of the seven-item scale: ‘Going abroad to get medical treatment in my opinion is:’ (−3 = harmful, +3 = beneficial; −3 = unpleasant, +3 = pleasant; −3 = bad, +3 = good; −3 = risky, +3 = safe; −3 = unprofitable, +3 = profitable; −3 = hard, +3 = easy; −3 = wrong, +3 = right). The direct subjective norms were assessed with one item: ‘The majority of the people important to me would approve of my travel abroad to get medical treatment’ −3 = definitely no, +3 = definitely yes). Finally, direct perceived control was also measured with one question: ‘If I considered going abroad to get medical treatment, I would be able to do it’ (−3 = definitely no, +3 = definitely yes). The wording of the questions was similar to what had been accepted in the research of Reddy et al. (2010) [33] (pp. 515–516), as they dealt with the intention to consider medical tourism instead of the actual travel. The measures are mostly reflective (direct), because the study focuses on predicting intention and not discovering the factors that provide the basis for the variables in the model [79]. The data are available at www.researchgate.net and can be found by DOI: 10.13140/RG.2.2.30188.72322.

The following online databases were analysed: EBSCO, ProQuest, Springer, Science Direct, Wiley, Scopus, and Web of Science; the following keywords were used: ‘Theory of planned behaviour’ and ‘medical tourism’, as well as the filter ‘scientific journals’ (peer reviewed) and text verification.

## 5. Data Analysis

As previously mentioned, all the items of the TPB model were measured with a 7-point Likert scale (from −3 to +3 with a middle value of 0). Therefore, the answers higher than zero indicate a generally positive outlook on medical tourism. The three components of the TPB model were measured directly (behavioural intention, subjective norms, perceived behavioural control). One component (attitude) was measured indirectly, using seven questions in the form of a semantic differential scale. To assess the reliability of this scale, Cronbach’s alpha was calculated [80], and the result was 0.77. This indicates good internal consistency as this value is higher than 0.6, which is often considered the minimum acceptable threshold [81]. However, a more detailed analysis of the correlation between the variables suggests that the first four questions of this scale constitute a more reliable measure of ‘attitude’, so only these four questions were used in further analysis (Cronbach’s alpha for this set amounted to 0.81).

The first two columns in Table 1 show the means and standard deviations of the variables in the TPB model (‘attitude’ is the average of the four aforementioned direct items). All the means are higher than zero (even higher than one), indicating a generally favourable outlook on medical tourism among the population being studied. The last row of Table 1 shows the statistics for ‘gender’ variable encoded as 1—male, 0—female. The average value of 0.53 means that 53% of respondents were men and 47% were women.

To verify the TPB model, a multiple regression model was estimated [82] in which the dependent variable is ‘behavioural intention’ and the independent variables are: ‘attitude’, ‘subjective norms’, and ‘perceived behavioural control’, with ‘gender’ added as the control variable. The results of the model are in the last four columns of Table 1. All three independent variables from the basic TPB model are statistically significant for behavioural intention to participate in medical tourism. In any case, this effect is positive. The strongest predictor is ‘attitude’ (*β* = 0.439, *t* = 7.92, *p* < 0.001); the two other components of the TPB model are less important. Gender proved to be an insignificant factor for behavioural intention to participate in medical tourism. All the exogenous variables in total significantly affect the explained variable (*F*_(4,516)_ = 55.49, *p* < 0.001), but the model generally explains a small fraction of the variability of ‘behavioural intention’ (Adjusted *R^2^* = 0.30).

One of the hypotheses (H4) proposed by the authors is that the TPB model parameters in different countries may be different. In order to verify the hypothesis, it was first decided to check whether the outlook on medical tourism is different between the countries examined. A Multivariate Analysis of Variance (MANOVA) was conducted (Warne [ref?]), with the dependent variables: ‘behavioural intention’, ‘attitude’, ‘subjective norms’, ‘perceived behavioural control’, and ‘country’ as the grouping variable. The analysis showed that there are significant differences in the average variable values between the countries (*Pillai* = 0.0075, approx. *F*_(8, 1032)_ = 5.02, *p* < 0.001). Subsequently, univariate ANOVAs were conducted [83], as well as a post hoc analysis using the Scheffé test [84]. The results of both analyses are presented in Table 2.

As the results of the univariate ANOVAs indicate, the respondents from the three countries surveyed do not differ significantly in average level of ‘behavioural intention’ and ‘attitude’ (p-values equal 0.063 and 0.196 respectively). However, ‘subjective norms’ and ‘perceived behavioural control’ differ significantly (*p*-values equal 0.045 and 0.004, respectively). In both cases, the differences are most significant between Poland and Turkey. The letters resulting from the Scheffé test in the last column of Table 2 indicate whether there are significant differences in any pair of means (within one variable). The occurrence of the same letter in two rows indicates no significant difference (a significance level of α = 0.05 is assumed for each test). In the case of ‘subjective norms’, the average level of the variable is significantly lower in Poland than in Turkey, i.e., the respondents in Turkey report greater approval for medical tourism by their relatives and friends than their counterparts in Poland. However, in the case of ‘perceived behavioural control’, the situation is reversed. In Poland, the average value of the variable is significantly higher than in Turkey, so the respondents from Poland perceive their ability to participate in medical tourism as more real. For both variables, the average values for Jordan are between those for Poland and Turkey, without statistically significant differences. Overall these results show that the average levels of TPB components may be different between countries, which in some way may (but does not need) affect the relationships in the TPB model.

More relevant than the differences in the average levels of the variables examined, however, are the potential differences in the effects of the exogenous variables from the TPB model on behavioural intention to participate in medical tourism. To check if these differences occur, multiple regression models, the same as for the entire sample, were estimated separately for each country. The estimated model parameters are shown in Table 3.

The model parameters estimated separately for each country differ between countries and are different from those in the combined model in each case. For example, perceived behavioural control proved to be an insignificant predictor of behavioural intention in Turkey and Jordan, yet highly significant in Poland. The strongest predictors of behavioural intention to participate in medical tourism are respectively: attitude (Jordan), subjective norms (Turkey), and attitude and perceived behavioural control (Poland) (the effect of this second variable is lower-lower regression coefficient *β*, but more stable-higher *t* statistic). Also, the models fit the data differently-adjusted *R^2^* for models in Poland, Turkey, and Jordan are 0.44, 0.24, and 0.25, respectively.

The observed differences in regression coefficients between the countries seem large, but it is worth checking to see if they are statistically significant. To do this, the following combined model with interactions was estimated:

(1) $$BN_{i}=\beta_{0}+\beta_{1}{\mathrm{AT}}_{i}+\beta_{2}{\mathrm{SB}}_{i}+\beta_{3}{\mathrm{PC}}_{i}+\beta_{4}{\mathrm{Sx}}_{i}+\beta_{5}C_{i}+\;\;\;\;\;\;\;\;\;\;\;\;\;\;\;+\beta_{6}C_{i}{\mathrm{AT}}_{i}+\beta_{7}C_{i}{\mathrm{SB}}_{i}+\;\beta_{8}C_{i}{\mathrm{PC}}_{i}+\beta_{9}C_{i}{\mathrm{Sx}}_{i}+e_{i}$$

where: *BN*_i_—behavioural intention, *AT_i_*—attitude, *SB_i_*—subjective norms, *PC_i_*—perceived behavioural control, *Sx_i_*—gender (male/female), *C_i_*—country (Poland/Turkey/Jordan), *e_i_*—error term.

The model notation is simplified because the variable actually consists of two dummy variables for two of the three countries. In addition, to obtain all three possible comparisons for the three groups/countries, two models were estimated, with different reference countries, so all the parameters for the ‘country’ variable (from *β*_5_ *to β*_9_) have three variants, each for a pair of countries. The significance of the differences between the countries in the regression coefficients shown in Table 3 is the same as the significance of the corresponding coefficients in terms of the interaction with the ‘country’ variable. In the context of this study, the coefficients *β*_6_, *β_7_*, and *β*_8_ are of interest, and the estimates for each pair of countries are shown in Table 4. In addition, the estimates of *β*_9_ (which illustrate the differences in the effect of gender) are also shown.

The results presented in Table 4 clearly indicate that the parameters of the TPB-based model are substantially different across the countries surveyed. For the three explanatory variables in the model (‘attitude’, ‘subjective norms’, and ‘perceived behavioural control’) and the three pairs of countries, which gives a total of nine parameters, the differences are statistically very significant in as many as four cases (*p* < 0.01), and fairly significant in one additional case (*p* = 0.059). For example, the influence of attitude is much higher in Jordan than in Turkey (*β* = 0.370, *p* = 0.005), while the effect of subjective norms is much higher in Turkey than in Poland (*β* = 0.320, *p* = 0.005). The significant differences in regression coefficients between the countries show that when modelling ‘behavioural intention to participate in medical tourism’, it is necessary to do it within a country or to take the country’s interactions with the other explanatory variables in the TPB model into account.

The adjusted *R^2^* for the abovementioned full model with interactions is only 0.33. The coefficients of determination in the previous models are also at unsatisfactorily low levels. Overall, the three components of the TPB model have a strong positive impact on behavioural intention, but even after the differences in the effects between the countries are taken into account, there remains a very large portion of variability in terms of ‘behavioural intention’, which is due to factors not included in the model. Perhaps the model does not include all the relevant exogenous variables; thus the TPB model is incomplete.

Summing up the above results, respondents from the three countries surveyed are generally positive about medical tourism. On average they would consider it, if they had such a possibility. Their attitudes towards it are closer to terms beneficial, pleasant, good, and safe, rather than harmful, unpleasant, bad, and risky, respectively. Young consumers’ behavioural intension to participate in medical tourism is driven by their attitudes, subjective norms, and perceived behavioural control, with the first predictor being the strongest one. All the explanatory variables positively affect ‘behavioural intension’. These effects are positive in all studied countries but their sizes differ. Variable ‘attitude’ is the strong predictor in Jordan and Poland, ‘subjective norms’ matter most in Turkey, and ‘perceived control’ plays the most significant role in Poland. Much of the variability in ‘behavioural intension’ is not explained by variables defined in TPB. Gender is not of much importance in any tested model.

## 6. Discussion

The analysis of the data shows that, in the segment studied, all the explanatory variables (‘attitude’, ‘subjective norms’ and ‘perceived behavioural control’) have a significant impact on the explained variable, that is, the intention to use medical tourism services. The results are similar to those obtained by other researchers, but some important differences occurred as well.

Taking all the respondents from the three countries collectively, the most significant predictor is attitude—which is consistent with studies of the intention to use medical tourism services conducted by Seow et al. [29] and Martin et al. [26], as well as studies of the willingness to consider using medical tourism services in developing countries by Reddy et al. [33]. There are more studies confirming the relationship between positive attitudes towards medical tourism (although it was not the most significant factor) and the intention to use medical services abroad for the second time [28] and to choose a destination providing health care services in accordance with Sharia law [32]. The factor turned out to be insignificant in only one study conducted by Ramamonjiarivelo et al. [34].

In this study, the second most important variable was ‘subjective norms’–which is consistent with the findings by Reddy et al. [33], Martin et al. [26], Seow et al. [29], and inconsistent with the studies by Lee et al. [27], Martin et al. [26] and Ramamonjiarivelo et al. [34], in which it had the strongest effect. The factor turned out to be insignificant as regards the choice of a destination providing health care services in accordance with Sharia law [32].

Perceived behavioural control has a smaller, yet statistically significant, effect as well—which is consistent with studies by Martin et al. [26] and Ramamonjiarivelo et al. [34]; and inconsistent with studies by Seow et al. [29] and Reddy et al. [33]. However, in a study by Suki et al. [32], the factor turned out to be the most significant one.

Similarly, mixed results were obtained in 15 studies of a different aspect of tourism, that is, intentions pertaining to the choice of a travel destination [85]. However, as the author of the TPB states, these results do not disconfirm the theory, because their significance may vary depending on the behaviours and populations studied, while the lack of predictive validity of one of the factors only means that it is not important in shaping the intention in a given context [79].

It is worth noting that attitude turned out to be the most significant factor in other studies with subject samples comprising students, i.e., Martin et al. [26] and Reddy et al. [33]. Seow et al. [29], whose study involved mostly young respondents, claimed it was due to their being more eager to experience new things.

In this study, subjective norms and perceived behavioural control were less significant. As far as the first factor goes, it can be due to the individualistic tendencies and the resistance to external pressure demonstrated by young people [29]. As for the second factor, its lower significance can be the result of the perceived ease of travelling in today’s globalised world, as well as lower awareness of the problems involved in organising such travels among young people, who usually have not faced health challenges in their lives to date. Of course, the aforementioned assumptions require further research.

The results of the study show that all respondents have positive attitudes towards medical tourism. Contrary results were obtained by Ramamonjiarivelo et al. [34] and Reddy et al. [33]. However, the latter study concentrated on participating in medical tourism in developing countries only, not medical tourism per se.

Moreover, the study shows significant differences in the levels of subjective norms and perceived behavioural control between Poland and Turkey. In each case, a favourable outlook on medical tourism is observed (the average of answers is above 0—the mid-point). However, the mean of variable ‘subjective norms’ is significantly lower in Poland than in Turkey, which means that respondents in Poland do not see as much approval for medical tourism among their relatives and friends as do the respondents in Turkey.

The opposite is true for perceived behavioural control. In Poland, the average value of the variable is significantly higher than in Turkey, which means that respondents from Poland perceive their ability to participate in medical tourism as more real. It may be due to the fact that Poles are free to travel in all the member states of the EU, but citizens of Turkey require visas to do so, which may influence their perception of travel abroad in general.

The research shows that the effect of the individual variables on the behavioural intention to participate in medical tourism differs depending on the respondents’ country of origin. The strongest predictors of the behavioural intention to participate in medical tourism are respectively: attitude (Jordan), subjective norms (Turkey), and attitude and perceived behavioural control (Poland), whereby perceived behavioural control proved to be an insignificant predictor of behavioural intentions in Turkey and Jordan, and a highly significant one in Poland. The discrepancies may be accounted for by social, economic, and legal circumstances in the countries; but in order to confirm the hypothesis, more detailed analyses are required.

Although there are studies confirming that the gender of medical tourists affects their behaviour in the context of the choice of destination and the type of medical tourism product [86,87], this variable was not analysed in the context of medical tourism and the TPB. In this study, it turned out to be an insignificant factor for the behavioural intention to participate in medical tourism.

The data analysis shows that in the segment studied, the TPB model may be incomplete, which means that it does not include all the exogenous variables. However, Ajzen (n.d.) [79] points out that, in principle, the TPB model is open and additional predictors may be included. Therefore, the variables should be identified in further studies.

## 7. Conclusions

The study of the applicability of the TPB model in medical tourism has been conducted among potential future medical tourists from Poland, Jordan, and Turkey. Upon analysing the data obtained, all the hypotheses proposed by the authors were confirmed, namely:

- there is a relationship between the intention to participate/consider participation in medical tourism and the three TPB factors (attitudes towards participation in medical tourism, subjective norms, and perceived behavioural control);
- the effect of the individual TPB factors on behavioural intentions as to medical tourism may vary among consumers from different countries.

It was shown that, for the population studied, all three explanatory variables have a significant effect on the explained variable, i.e., the intention to use medical tourism services. The strongest predictor was attitude, followed by subjective norms and perceived control. Hence, the analysis has shown that the TPB model can be applied to medical tourism in the segment of young consumers who were the subjects of research.

As far as the potential differences between subjects from different countries go, the analysis has shown that the country of origin is most significant in the case of two variables, i.e., subjective norms and perceived control. No such effect was identified in terms of attitudes towards medical tourism and the very intention to participate/consider participation in medical tourism (the respondents from the three countries surveyed have a similar average level of behavioural intention and attitude). It must be noted, however, that the country of origin not having a significant influence on two other variables, namely attitude and intention, does not mean that the country of origin is not an important variable in the context of the TPB in the sample studied. The strongest predictor of behavioural intention among the respondents from Jordan was attitude; in Turkey, it was subjective norms; and in Poland, attitude and perceived control.

The analysis of the data has also shown that, in the segment studied, the TPB model may be incomplete, which means that it does not include all the exogenous variables. There are most probably other variables which could be added to the model, and the intention to participate/consider participation in medical tourism requires a more detailed description.

To sum up, the analysis shows that young consumers from the three countries have positive attitudes towards medical tourism. This may have considerable implications for companies rendering transborder services in medical tourism—in particular, for their long-term growth and expansion strategies.

With the aforementioned relationship between countries of origin and consumers’ intention to use a service, companies planning to enter new markets abroad need to consider making adjustments in their marketing activities to allow for the differences between their clients from diverse countries. In the medical tourism sector, uniform marketing campaigns may be less effective than campaigns tailored to local/regional market conditions.

### Limitations and Future Studies

The selection of the sample was non-probabilistic, which means that we should be careful when extrapolating the results onto the entire population. However, the subject of the inference is the relationships between the variables, which are usually less affected by a sample selection mechanism. Moreover, the recruitment procedure was the same in all three countries, which decreases the risk of bias in the comparison made between the countries.

The study was conducted among young consumers. While the results may be interesting, they refer to the young generation of consumers and they should not be extrapolated on other age groups in the countries studied. With very few studies and publications on the TPB in medical tourism, this study should be treated as an initial analysis of the phenomenon and the first stage of a more comprehensive research process involving consumers from different countries and age groups.

Taking into account the incompleteness of the TPB model in the young consumer segment in the countries studied, more detailed qualitative research should be conducted among actual medical tourists to determine some additional variables in the model.

## Figures and Tables

**Table 1:** Descriptive statistics and multiple regression results (dependent variable: behavioural intention).

Dependent Variable	Descriptive Statistics		Multiple Regression Results			
	Mean	Std. dev.	Coefficient	Std. Error	*t*	*p*-Value
Behavioural intention	1.20	1.57	-	-	-	-
(Intercept)	-	-	0.299	0.106	2.81	0.005
Attitude	1.07	1.5	0.439	0.055	7.92	0.000
Subjective norms	1.09	1.1	0.206	0.044	4.64	0.000
Perceived control	1.12	1.4	0.191	0.044	4.38	0.000
Gender (male)	0.53	-	−0.029	0.117	0.25	0.804

Source: Own calculations based on survey results.

**Table 2:** Descriptive statistics, ANOVA, and post hoc analysis for TPB components, by country.

TPB Component	Country	*n*	Mean	Std. dev.	ANOVA	Groups Based on Scheffé Test *
Behavioural intention	Poland	213	1.39	1.53	*F*_(2, 518)_ = 2.78, *p* = 0.063	-
	Turkey	125	1.08	1.55		-
	Jordan	183	1.05	1.61		-
Attitude	Poland	213	1.17	1.13	*F*_(2, 518)_ = 1.64, *p* = 0.196	-
	Turkey	125	1.08	1.28		-
	Jordan	183	0.96	1.08		-
Subjective norms	Poland	213	0.93	1.51	*F*_(2, 518)_ = 3.12, *p* = 0.045	b
	Turkey	125	1.34	1.58		a
	Jordan	183	1.12	1.43		ab
Perceived control	Poland	213	1.32	1.51	*F*_(2, 518)_ = 5.55, *p* = 0.004	a
	Turkey	125	0.75	1.68		b
	Jordan	183	1.14	1.43		ab

* means with the same letter are not significantly different Source: Own calculations based on survey results.

**Table 3:** Multiple regression results by country (dependent variable: behavioural intention).

Dependent Variable	Country	Coefficient	Std. Error	*t*	*p*-Value
(Intercept)	Poland	0.156	0.159	0.98	0.328
	Turkey	0.436	0.231	1.89	0.062
	Jordan	0.280	0.184	1.53	0.129
Attitude	Poland	0.430	0.091	4.70	0.000
	Turkey	0.219	0.097	2.25	0.027
	Jordan	0.588	0.102	5.80	0.000
Subjective norms	Poland	0.086	0.066	1.31	0.193
	Turkey	0.406	0.091	4.46	0.000
	Jordan	0.191	0.080	2.40	0.018
Perceived control	Poland	0.374	0.069	5.42	0.000
	Turkey	0.016	0.084	0.19	0.849
	Jordan	0.086	0.079	1.09	0.277
Gender (male–female)	Poland	0.239	0.166	1.44	0.152
	Turkey	−0.287	0.246	−1.16	0.247
	Jordan	−0.275	0.215	−1.28	0.202

Source: Own calculations based on survey results.

**Table 4:** Comparisons of coefficients in multiple regression (dependent variable: behavioural intention).

Dependent Variable	Comparison	Coefficient	Std. Error	*t*	*p*-Value
Attitude	Turkey-Poland	−0.211	0.138	−1.53	0.127
	Jordan-Poland	0.159	0.139	1.14	0.254
	Jordan-Turkey	0.370	0.132	2.80	0.005
Subjective norms	Turkey-Poland	0.320	0.114	2.81	0.005
	Jordan-Poland	0.105	0.104	1.01	0.315
	Jordan-Turkey	−0.215	0.114	−1.89	0.059
Perceived control	Turkey-Poland	−0.358	0.111	−3.22	0.001
	Jordan-Poland	−0.288	0.106	−2.71	0.007
	Jordan-Turkey	0.070	0.108	0.65	0.518
Gender (male–female)	Turkey-Poland	−0.525	0.299	−1.76	0.080
	Jordan-Poland	−0.513	0.272	−1.89	0.060
	Jordan-Turkey	0.012	0.307	0.04	0.969

Source: Own calculations based on survey results.
